# Synapsin III in brain development

**DOI:** 10.18632/oncotarget.8106

**Published:** 2016-03-16

**Authors:** Laura E. Perlini, Fabio Benfenati, Laura Cancedda

**Affiliations:** Department of Neuroscience and Brain Technologies, Istituto Italiano di Tecnologia, Genova, Italy

**Keywords:** Synapsin III, semaphorin 3A, CDK5, Reelin, Src-family kinases

Since the dawn of neuroscience, genetic alterations causing macroscopic brain defects have attracted the attention of scientists. However, in recent years, the increasing understanding of the biological causes of neurological disorders such as Autism Spectrum Disorder or Schizophrenia have indicated that affected patients do not display *gross* structural alterations in the brain. These findings have shifted the attention of scientists toward the study of genetic alterations that may cause *subtle* defects in brain architecture. The investigation on such microscopic brain alterations is currently favored by the progress in experimental techniques for brain research. In Perlini et al., 2015 [1], we took advantage of the *in utero* electroporation technique coupled with RNA interference to examine the role of Synapsin III (SynIII) in the developing neocortex.

SynIII belongs to the Syn family, which comprises three phosphoproteins abundantly expressed in the brain. Whereas a large body of literature indicates that both SynI and SynII are mainly involved in synaptic transmission and plasticity in the adult brain, the available data suggest an involvement of SynIII in brain development. First, SynIII is expressed early during mammalian brain formation and is involved in early stages of neuronal development *in vitro*, while it is downregulated in the adult brain [2, 3]. Second, SynIII participates in adult neurogenesis [3], and often proteins involved in adult neurogenesis are also players in brain development. Third, SynIII SNPs have been implicated in neurodevelopmental disorders such as schizophrenia, and SynIII knockout (KO) mice display some behavioral traits of schizophrenia [4]. Finally, according to evolutionary studies, the Syn family originated from a single primordial *Syn* gene that was expressed in the brain since early development [5]. As in higher vertebrates SynI and II are expressed mostly in the adult brain, SynIII probably carries out the functions performed during development by the primitive single Syn. Interestingly, the emergence of a distinct *SynIII* gene correlates with the appearance of the telencephalic region during evolution [6].

Despite the evidence suggesting a developmental role for SynIII, the initial analysis of SynIII KO mice revealed a mild behavioral phenotype with no gross alterations in brain development. We hypothesized that the alterations caused by SynIII absence could be very subtle and difficult to detect with conventional histochemical techniques. In Perlini et al., 2015 [1], we demonstrated that SynIII KO brains do have aberrant brain development with defects in neuronal orientation as visualized by *in utero* transfection of a fluorescent (GFP) reporter. As the migration and orientation phenotypes were milder in SynIII KO mice than in animals where SynIII was acutely downregulated by RNA interference, we hypothesized that a partial compensation phenomenon could be triggered by the chronic depletion of the protein in KO animals. Interestingly, also animals with *in utero* upregulation of SynIII levels presented defects in radial migration with the appearance of ectopic cells at late stages. Thus, SynIII plays a key role in the orchestration of cortical development, and a tight regulation of its expression is essential for this function.

In Perlini et al., 2015 [1], we also demonstrated that the migration and orientation defects upon SynIII depletion involved the Sema3A/CDK5 signaling cascade. Indeed, Sema3A stimulation caused SynIII phosphorylation by CDK5 on Ser^404^ and such CDK5-dependent phosphorylation was essential for Sema3A-induced SynIII activity. It would be interesting to investigate whether SynIII participates in other developmental events mediated by Sema3A. Indeed, Sema3A is also involved in axon/dendrite initiation during the early phase of neuronal polarization through its negative regulation of PKA activity. Interestingly, SynIII also carries a highly conserved phosphorylation site targeted by PKA (Ser^9^), and defects in neuronal polarization consequent to SynIII downregulation were recently observed [7].

Besides the findings in Perlini et al., 2015 [1] demonstrating that SynIII acts downstream Sema3A, the existence of multiple phosphorylation sites on SynIII indicates that SynIII may be potentially downstream to other signaling pathways. Interestingly, during development, migrating cortical neurons encounter an increasing gradient of both Sema3A and Reelin around the marginal zone, and mice defective in Sema3A- and Reelin-signaling share common phenotypic features, such as misoriented neurons, also found in SynIII-downregulated brains. This suggests that Sema3A/SynIII and Reelin signaling interact at least in part. Notably, this interaction might be important for proper brain development and function, as an increase in Sema3A with a concomitant decrease of Reelin was observed in brains from schizophrenic patients. Moreover, both Sema3A and Reelin activate the Src-family kinase Fyn. The presence of a phosphorylation site for Src-family kinases (Tyr^301^) on SynIII puts this protein again downstream of Sema3A, but potentially also downstream of Reelin signaling, as a potential link through these two pivotal pathways for brain development. Therefore, despite the recent advancements in the comprehension of the role of SynIII in brain development, many questions on other potential functions of this protein remain open.
